# Acceptance and Commitment Training for Family Caregivers of People with Neurodevelopmental Disabilities: Protocol for a Collaborative Implementation Study

**DOI:** 10.2196/75049

**Published:** 2025-12-04

**Authors:** Kenneth Fung, Yona Lunsky, Kendra Thomson, Lee Steel, Soumya Mishra, Jodie Siu, Nicole Bobbette, Carly Magnacca, Jonathan Weiss, Melanie Penner, Ullanda Niel, Dorothee Chopamba, Sheila Phillips, Avra Selick, Brianne Redquest, Johanna Lake

**Affiliations:** 1 Department of Psychiatry Temerty Faculty of Medicine University of Toronto Toronto, ON Canada; 2 Centre for Mental Health Toronto Western Hospital Toronto, ON Canada; 3 Azrieli Adult Neurodevelopmental Centre Centre for Addiction and Mental Health Toronto, ON Canada; 4 Department of Applied Disability Studies Brock University St. Catharines, ON Canada; 5 School of Rehabilitation Therapy Queen's University Kingston, ON Canada; 6 Department of Psychology York University Toronto, ON Canada; 7 Autism Research Centre Holland Bloorview Kids Rehabilitation Hospital Toronto, ON Canada; 8 Department of Paediatrics Temerty Faculty of Medicine University of Toronto Toronto, ON Canada; 9 Scarborough Centre for Healthy Communities Toronto, ON Canada; 10 Department of Family and Community Medicine Temerty Faculty of Medicine University of Toronto Toronto, ON Canada; 11 Department of Family Medicine Queen's University Kingston, ON Canada

**Keywords:** Acceptance and Commitment Training, caregivers, neurodevelopmental disabilities, peer facilitation, implementation science, adaptation, equity

## Abstract

**Background:**

Family caregivers of individuals with neurodevelopmental disabilities (NDDs) often experience stress, anxiety, and depression; however, few evidence-based interventions are designed to improve their mental well-being. To address this gap, we developed an acceptance and commitment training (ACT) group–based workshop cofacilitated by trained caregivers and clinicians (Caring for the Caregiver Acceptance and Commitment Training [CC-ACT]).

**Objective:**

This study evaluates the real-world implementation of this innovative, evidence-based ACT workshop aimed at enhancing caregiver mental health and resilience.

**Methods:**

Guided by the reach, effectiveness, adoption, implementation, and maintenance (RE-AIM) implementation science framework, this study examines the workshop across these 5 domains. We delivered the CC-ACT workshops virtually or in-person across 11 intervention sites in Canada, including hospital and community agencies that provide services to children with NDDs and their families. Family caregivers (ie, a parent, grandparent, or adult sibling) of someone with an NDD were eligible to participate in the workshops, with site-specific criteria set by each host agency. Caregivers participated in preintervention, postintervention, and 3-month follow-up assessments measuring stress, resilience, and self-compassion using validated instruments (21-item Depression, Anxiety and Stress Scale; Parenting Stress Index, 4th Edition; Brief Family Distress Scale; Multi-System Model of Resilience Inventory; and Self-Compassion Scale–Short Form), alongside ACT process measures (Cognitive Fusion Questionnaire, Valued Living Questionnaire, and Acceptance and Action Questionnaire-II). Implementation fidelity was assessed through checklists and surveys. Focus groups with caregiver facilitators, clinician facilitators, workshop participants, and organizational leaders were held to qualitatively evaluate the implementation process and the caregiver-clinician cofacilitation model. Qualitative data will be analyzed using descriptive content analysis, a flexible approach that can be used to systematically summarize different types of qualitative data. Quantitative data will be analyzed through repeated measures ANOVA and mixed-effects modeling, with subgroup analyses and multiple imputation for missing data.

**Results:**

The CC-ACT workshops successfully reached 195 caregivers of individuals with NDDs. Two focus groups that included 5 caregiver workshop participants, 13 facilitators, and 5 organizational leaders were conducted. We anticipate that the workshops will demonstrate positive impacts on caregiver well-being, with variability in effectiveness based on participant characteristics and real-world implementation contexts. The findings are expected to identify key predictors of outcomes, equity and access barriers, and best practices for scaling and sustaining high-fidelity, adaptable caregiver interventions across diverse Canadian settings. Funding began in January 2022, data collection was completed in 2024, and data analyses will be completed by the end of 2025.

**Conclusions:**

The CC-ACT workshop is a promising approach to enhancing the mental well-being of caregivers of individuals with NDDs. The RE-AIM framework helps capture process data systematically, documenting the balance between fidelity and adaptation. The study findings should support the refinement of implementation strategies and support the broader scalability of the intervention to diverse community settings.

**International Registered Report Identifier (IRRID):**

DERR1-10.2196/75049

## Introduction

### Background

Family caregivers (hereafter referred to as caregivers) of individuals with neurodevelopmental disabilities (NDDs) report high levels of stress, anxiety, and depression [1-3]. Their vulnerability was highlighted during the COVID-19 pandemic, when they had to navigate the sudden loss of structure, support, and routine for their loved ones [4]. Unfortunately, few evidence-based interventions are available to support the well-being and resilience of caregivers. Furthermore, these programs are limited in scope (eg, restrictions around age or disability of the individual being cared for), or in availability (eg, only in regions with extensive resources) [5,6].

Caregiver interventions help support the caregiver and, by extension, the entire family, including the child with a disability or neurodevelopmental condition [7]. While caregivers may benefit from individual support, group-based and peer-led interventions may confer additional benefits, address feelings of isolation and provide opportunities for personal growth, connection, and mutual learning [8,9]. Typically, these interventions are delivered by trained clinicians [10]; however, there is emerging evidence that caregivers can also play an important role as peer cofacilitators, ultimately empowering caregivers’ voices while enhancing the success and feasibility of interventions [11].

To support caregivers of children with autism, early evidence demonstrated the benefits of acceptance and commitment training (ACT), a mindfulness-based workshop [12], as well as a study demonstrating the engagement of mothers as peer mentors in delivering a mindfulness-based intervention [13]. On the basis of our previous work using ACT in nonclinical populations to promote well-being and address issues related to stigmatizing attitudes, we adapted a 2-day in-person group-based ACT workshop for caregivers of children with autism to increase their resilience in the face of stressors, co-delivered by caregivers and clinicians together [14,15].

Early efficacy research demonstrated that our in-person ACT workshop was effective in reducing stress, depression, physical health concerns, and social isolation in mothers of children with autism and youth [16]. Additionally, the workshop helped these caregivers detach themselves from their distressing thoughts and act in ways that were more consistent with their values [14]. In a follow-up randomized controlled trial (RCT), caregivers of individuals with autism who participated in the workshop showed significant improvements in symptoms of depression and family distress relative to a waitlist control group, as well as significant treatment group improvements in stress, psychological acceptance, and cognitive fusion, which were not found in the waitlist group [17]. Workshop respondents also reported that a key element to the success of the workshops was the caregiver-clinician partnership. We have started to explore the use of our workshop for caregivers of other NDDs, such as individuals with fetal alcohol spectrum disorder (FASD) [18]. With the onset of the pandemic, we adapted our in-person group workshop into a virtual group workshop. Virtual delivery also addressed access barriers, such as a lack of childcare, distance from services, and transportation costs.

### Research Objectives

The aim of this study is to evaluate the community implementation of the Caring for the Caregiver ACT (CC-ACT) model, designed to enhance the mental health and resilience of caregivers of individuals with NDDs, co-delivered by caregiver-clinician facilitation teams. Our research objectives and questions are presented in Textbox 1.

**Research objectives and questions.**

1. Evaluate the implementation of a caregiver and clinician-partnered Caring for the Caregiver Acceptance and Commitment Training (CC-ACT) workshop delivered in communities across Canada to support the mental health and resilience of caregivers of individuals with neurodevelopmental disabilities.
  Is it feasible to recruit caregiver participants across study sites, and what proportion of participants complete the workshop?
  Does the workshop lead to enhanced mental health and resilience in participants, and is this change maintained at follow-up?
  What demographic, clinical, and organizational variables predict or moderate change, for example, whether the workshop is delivered to a mixed or homogeneous disability caregiver group, delivered in-person or virtually, or to caregivers of children or young adults?
  How is the workshop perceived by various community groups, including participants, clinician facilitators, caregiver facilitators, and organizational leaders?
  What adaptations become necessary as the workshop is implemented in various real-world settings?
  What is the long-term sustainability of the workshop program in terms of its resource use, time, and potential for wide-scale adoption?
2. Evaluate the unique collaboration between caregivers and clinicians codelivering the workshop.
  What are the facilitators and barriers to successful caregiver-clinician collaborations with regard to facilitator training and workshop delivery?
  What is the value of this collaboration from various community group perspectives, including participants, clinician facilitators, caregiver facilitators, organizational leaders, and the research team?

## Methods

### Implementation Science Framework: Reach, Effectiveness, Adoption, Implementation, and Maintenance

The study is guided by the reach, effectiveness or efficacy, adoption, implementation, and maintenance (RE-AIM) implementation science framework [19], providing an overarching structure to examine the processes and outcomes of the real-world implementation of evidence-based health workshops in 5 areas: reach, effectiveness, adoption, implementation, and maintenance. *Reach* is a measure of participation at the individual level, such as the number or characteristics of program participants. *Effectiveness* includes an assessment of the positive and negative outcomes of the program. *Adoption* examines the uptake at the individual or organizational level. *Implementation* examines the degree to which the program is conducted as intended in various real-world settings. *Maintenance* evaluates the extent to which the program impact is enduring at the individual and organizational levels.

### CC-ACT Workshop Description

Our workshop is a group intervention based on ACT, which aims to increase psychological flexibility through training in 6 core processes: defusion (relating to thoughts as merely thoughts), acceptance (willingness to experience thoughts and feelings rather than avoiding them), contact with the present moment (being in touch with the here and now rather than being stuck in the past or worrying about the future), self-as-context (being in contact with a transcendent sense of self rather than being stuck with self-labels), values (clarifying what gives life value and meaning), and committed action (persisting in value-guided actions). The intervention consists of experiential exercises, mindfulness practices, and small and large group reflective sharing [20].

Groups are guided by trained cofacilitators, including at least one clinician with experience in the NDD community and at least one family caregiver of an individual with an NDD. Each group can accommodate 10 to 15 participants. For in-person delivery, the workshop is delivered over 2 consecutive sessions, an evening session (3 h) and a full-day session (8 h), followed by a final evening session (3 h) 1 month later (Table 1; refer to Fung et al [20] for more details on intervention content). For virtual delivery, the workshop is conducted over 4 to 5 consecutive weekly 2-hour sessions, with an additional 2-hour session occurring 1 month later.

**Table 1:** Overview of Caring for the Caregiver Acceptance and Commitment Training (CC-ACT) content across delivery modalities.

Virtual intervention (5 to 6 sessions; 2 h per session)	In-person intervention (3 sessions: S1 half-day [3 h], S2 full day [8 h], and S3 half-day [3 h])
S1: Overview of ACT (eg, hexaflex and hand gestures) Contact with the present moment exercise (eg, meditation with self-object, leaves on a stream)	S1: Overview of ACT (eg, hexaflex and hand gestures) Contact with the present moment exercise (eg, meditation with self-object and leaves on a stream)
S2: Acceptance exercise (eg, keeping the count)	S2: Acceptance exercise (eg, chair sculpture) Defusion exercise (eg, paired sharing) Values exercise (eg, values wordlist and 100th birthday) Committed action exercises (eg, bull’s eye and bus driver)
S3: Defusion exercise (eg, adventures of the pink elephant and our song)	—^a^
S4 (or S4 and S5): Values exercise (eg, values wordlist and 100th birthday) Committed action exercises (eg, bull’s eye and fireside chat)	—^a^
After a 4-week break, S5 (or S6): Review of ACT model (eg, hand gestures and matrix) Self-as-context exercise (eg, who I am)	After a 4-week break, S3: Review of ACT model (eg, hand gestures and matrix) Self-as context exercise (eg, LE’GO)

^a^Not applicable.

### Project Description

The proposed project was conducted in 3 overlapping phases.

#### Phase 1: Preparation and Contextualization

In this initial phase, research ethics and institutional approvals were obtained. Community partner organizations and family advisers were engaged to refine the implementation process. Site-specific discussions helped identify local needs and preferences, including in-person versus virtual delivery and whether to offer mixed or homogeneous caregiver groups (eg, caregivers of individuals with autism, FASD, Down syndrome, or spina bifida). A core implementation protocol and a fidelity checklist were developed to evaluate adherence and document adaptations. Each site completed the Health Equity Impact Assessment (HEIA) tool [21], a structured implementation support tool, to identify potential barriers to accessing or participating in the workshop for vulnerable populations and mitigation strategies. Facilitator teams—composed of experienced caregivers and clinicians—were trained and supported based on the specific needs of each site.

#### Phase 2: Implementation

From 2022 to 2024, we partnered with 11 sites to deliver up to 2 ACT workshops per site, with each workshop targeting 10 to 15 caregiver participants. Sites across Canada included hospitals and community agencies providing services for children or families with NDDs (Table 2). Facilitators at each of the sites completed 20 hours of basic training from our team. Caregivers were eligible to participate in the workshops if they identified as a family caregiver (ie, parent, grandparent, or adult sibling) of someone with an NDD. Depending on the mandate of the host agency, workshops were held for caregivers of people of specific ages (ie, children, youth, or adults) or specific NDD diagnostic groups (ie, autism or FASD). Workshops were conducted in 2 waves (6-9 mo apart) to allow learnings from the first wave to inform the second and ensure adequate recruitment time.

**Table 2:** Implementation sites.

Organization	Organization type	Province	Target population	Workshop format
1	Mental health hospital	Ontario	Two populations: caregivers of people aged >16 y with NDDs^a^ and fathers of people aged >16y with NDDs	5-wk virtual
2	Community health center	Ontario	Caregivers of children, youth, and adults with NDDs	5-wk virtual
3	Nonprofit organization: IDD^b^	Ontario	Caregivers of children, youth, and adults with NDDs	6-wk virtual
4	Nonprofit organization: sibling disabilities	Canada	Adult sibling caregivers of people with NDDs	6-wk virtual
5	Rehabilitation hospital	Ontario	Caregivers of children and youth with disabilities and complex health conditions	6-wk virtual
6	Nonprofit organization: disabilities	British Columbia	Caregivers of children and youth with NDDs and complex health conditions in early intervention and school-age programs	6-wk virtual
7	Nonprofit organization: disabilities	Ontario	Caregivers of children and youth with NDDs	6-wk virtual
8	Nonprofit organization: IDD	Quebec	Caregivers of children, youth, and adults with NDDs	6-wk virtual
9	Nonprofit organization: disabilities	Ontario	Caregivers of children, youth, and adults with NDDs	6-wk virtual
10	Nonprofit organization: developmental services	Ontario	Caregivers of individuals with fetal alcohol spectrum disorder	6-wk virtual
11	Community health center and nonprofit autism organization	Nova Scotia	Caregivers of people with autism	In-person

^a^NDD: neurodevelopmental disability.

^b^IDD: intellectual or developmental disability.

Experienced CC-ACT peer coaches provided virtual coaching to local facilitation teams as needed. After each ACT session, facilitators completed fidelity checklists noting the exercises they completed or did not complete and why, as well as describing how well the exercises worked and any ad hoc adaptations. After the workshop, facilitators completed a survey detailing their experiences delivering the intervention, the support they received (virtual coaching), and reflections on caregiver-clinician collaboration. CC-ACT peer coaches also completed a survey about their experience in coaching each of the local facilitation teams.

To evaluate effectiveness, caregiver participants completed electronic surveys at 3 time points: before intervention (T1), after intervention (T2), and 3-month follow-up (T3). The survey included demographic, outcome, and ACT process measures, as presented in Textbox 2.

**Demographic, outcome, and acceptance and commitment training (ACT) measures.**

**Demographic measures**

- Parent age, ethnicity, education, and family composition
- Child’s neurodevelopmental disability diagnosis, time since diagnosis, age, and living situation

**Outcome measures**

- Depression, Anxiety and Stress Scale: 21-item measure of depression, anxiety, and stress [22]
- Parenting Stress Index, 4th Edition: 11 items from Health and Isolation subscales to assess stress specific to parenting [23]
- Brief Family Distress Scale: Single-item 10-point scale for family distress [24]
- Multi-System Model of Resilience Inventory: 27-item scale assessing internal, external, and mediating resilience factors [25]
- The Short Warwick–Edinburgh Mental Well-being Scale: 7-item scale measured on a 5-point scale to assess mental well-being [26]
- Patient Health Questionnaire-4 (Kroenke et al [27]): 4-item scale for capturing anxiety and depressive symptoms [27]

**ACT process measures**

- Cognitive Fusion Questionnaire: 7-item scale assessing cognitive fusion, the degree to which individuals become entangled with their thoughts [28]
- Valued Living Questionnaire: 10-item 2-part tool evaluating value importance and alignment [29]
- Acceptance and Action Questionnaire-II: 7-item scale measuring psychological flexibility, the main target of acceptance and commitment training [30]
- Self-Compassion Scale–Short Form: 12-item measure of self-compassion [31]

Following the completion of the intervention, workshop participants were asked survey questions that measured their satisfaction with the CC-ACT program. Questions addressed the workshop content (how easy it was to understand, how relevant it was to their family, how interesting it was, and whether it contained new information), how much the workshop addressed the goals important to them, whether it provided skills that could be used in everyday life, the perceived value of having caregivers and clinicians colead the workshop, and how much the participants felt supported and valued throughout the workshop. Responses were provided on a 5-point Likert-type scale, ranging from strongly disagree (1) to strongly agree (5).

In addition, we conducted 3 focus groups, one each with caregiver workshop participants, facilitators, and organizational leaders, to explore perceptions of the intervention and caregiver-clinician collaboration (Multimedia Appendix 1).

#### Phase 3: Data Analysis and Knowledge Translation

##### Overview

In this final phase, we are analyzing quantitative and qualitative data. Lessons learned, particularly technical ACT-related facilitation tips, have been compiled into an implementation toolkit based on shared insights from facilitators and CC-ACT peer coaches. When we complete the analysis of the study outcomes and implementation data, we will publish and disseminate the findings to inform future intervention development and policy recommendations.

##### Project Evaluation Plan

The project is being evaluated using a mixed methods design to address the two primary research questions: (1) evaluation of CC-ACT intervention implementation using RE-AIM (Table 3) and (2) evaluation of caregiver-clinician collaboration (Textbox 3).

**Table 3:** Implementation process evaluation.

RE-AIM^a^ dimensions	Evaluation indicators and data sources
Reach	Number of people who expressed interest, number of people who were recruited, number of people who completed, and % of completers Sociodemographic and other participant characteristics (eg, type of NDD^b^) comparisons: Cross-site comparisons
Effectiveness	Before versus after comparisons: outcome measures; process measures Analysis of moderators on outcome measures Mediational analysis of process measures on outcomes Estimated cost ranges for staff, materials, and other items necessary to run the workshops Perceived benefits from cross-site focus groups
Adoption	Perceived feasibility, acceptability, and fit from focus groups
Implementation	Fidelity checklists Health Equity Impact Assessment Postworkshop surveys completed by facilitators and CC-ACT^c^ peer coaches
Maintenance	Before versus after versus follow-up survey data and focus groups Tracking of agencies implementing regular programming based on our model

^a^RE-AIM: reach, effectiveness, adoption, implementation, and maintenance.

^b^NDD: neurodevelopmental disability.

^c^CC-ACT: Caring for the Caregiver Acceptance and Commitment Training.

**Caregiver-clinician collaboration evaluation.**

**Evaluation Focus Areas**

- Barriers and facilitators to successful collaborations in terms of training and intervention delivery
- Stakeholder perspectives on the value of the collaboration

**Evaluation indicators and data sources**

- Postworkshop surveys completed by facilitators and Caring for the Caregiver Acceptance and Commitment Training peer coaches
- Perceived benefits of collaboration from cross-site homogeneous and mixed focus groups (eg, caregiver facilitators, clinician facilitators, workshop participants, and organizational leaders)
- Perceptions of the partnered model on the postintervention survey

### Ethical Considerations

#### Overview

The study was approved by the institutional research ethics board of our team’s mental health teaching hospital, the Centre for Addiction and Mental Health (REB#037-2017), and all participants provided informed consent online through REDCap (Research Electronic Data Capture; Vanderbilt University), an online survey platform, before commencing the study.

#### Surveys

Participation in the research evaluation was voluntary, and individuals could attend the workshops without taking part in the evaluation. Study data were collected and managed using REDCap electronic data capture tools hosted at the Centre for Addiction and Mental Health [32,33]. REDCap is a secure web-based software platform designed to support data capture for research studies, providing (1) an intuitive interface for validated data capture, (2) audit trails for tracking data manipulation and export procedures, (3) automated export procedures for seamless data downloads to common statistical packages, and (4) procedures for data integration and interoperability with external sources.

Eligible family caregivers and facilitators completed consent online through REDCap, directing them to preintervention (T1) surveys, including demographic information (eg, gender, race, and age). Postintervention (T2) and follow-up (T3) surveys were completed similarly and included repeated measures and qualitative reflection questions. Each survey took approximately 20 to 30 minutes for family caregivers and 10 to 20 minutes for facilitators to complete. Survey participants received honoraria in the form of a CAD $50 (US $35.74) e-gift card at the end of the data collection period.

#### Focus Groups

Workshop facilitators, organizational leadership, and workshop participants were invited to participate in separate virtual focus groups via email. Focus group participants participated in the group virtually using WebEx (Cisco Systems, Inc), a secure, web-based videoconference platform. The focus groups were recorded, transcribed verbatim, and anonymized. Focus group participants received honoraria in the form of a CAD $50 (US $35.74) e-gift card following their participation.

### Statistical Analysis

#### Quantitative Analysis

To evaluate reach, we will examine the number, attendance, and demographic profiles of participants at each site. To evaluate effectiveness and maintenance of the intervention, we will assess changes in outcome measures (21-item Depression, Anxiety and Stress Scale; Parenting Stress Index, 4th Edition; Brief Family Distress Scale; Multi-System Model of Resilience Inventory; Short Warwick–Edinburgh Mental Well-being Scale; and Patient Health Questionnaire-4) and ACT process measures (Cognitive Fusion Questionnaire, Valued Living Questionnaire, and Acceptance and Action Questionnaire-II) across 3 time points (before workshop, after workshop, and follow-up). We will first conduct a within-subjects repeated measures ANOVA, checking assumptions of normality (via skewness and kurtosis *z* values) and the absence of outliers (via boxplots). Where significant main effects are found, we will perform Bonferroni-adjusted pairwise comparisons (pretest-posttest, before follow-up, and after follow-up). In addition, we will use mixed-effects modeling to account for repeated measures within individuals and variability across study sites, and to examine potential predictors and moderators (eg, delivery modality, group composition, and demographic variables). Subgroup analyses will be stratified by gender identity, age, racial or ethnic identity, and living situation, exploring both the main effects and interactions. Missing data will be addressed according to the intention-to-treat principle, using multiple imputation in SPSS (IBM Corp) with all demographic and baseline outcome variables included as predictors.

#### Qualitative Analysis

The evaluation of all components of RE-AIM will also be undertaken using a qualitative approach. All qualitative data (focus groups, survey open-ended responses, and meeting notes) will be analyzed using descriptive content analysis [34]. This is a flexible approach that can be used to systematically summarize different types of qualitative data and is often used in applied research settings. Two team members will review the data and generate a codebook informed by the RE-AIM framework. Data will be independently coded, and reviewers will use a consensus. Several strategies will be used to establish trustworthiness for the qualitative components of this study, including credibility, dependability, transferability, and confirmability [35,36]. Data collection instruments (ie, focus group guide and open-ended survey questions) will be codeveloped by members of our multidisciplinary team, including caregivers and clinicians, to ensure they are clear, relevant, and easy to understand. We will create an environment in which participants feel comfortable expressing their opinions, including ensuring that focus groups are facilitated by an external individual who is not directly involved in delivering the intervention, and we will hold separate groups for caregivers, facilitators, and organizational leadership. An iterative coding process will be used based on multiple readings of the data and the perspectives of multiple team members. An audit trail will be used to clearly document each step of the analysis process. Thick description will be used in reporting to contextualize findings.

#### Data Management

Quantitative data were deidentified, and qualitative data were anonymized to safeguard participant confidentiality. Data are stored in a password-protected database on a secure computer network, accessible only to authorized research staff who will abide by the confidentiality regulations set by the Research Ethics Board. Each survey participant was assigned a unique ID number, which was used to link their data across all research activities and time points. The master list linking participant IDs to identifying information is stored in a separate, secure folder on the computer network.

Survey and focus group data are securely managed using a REDCap database designed and maintained for this study. Data are monitored and reviewed frequently by study investigators to ensure adherence to confidentiality protocols, Research Ethics Board guidelines, and protocol design. At the end of the study, all data will continue to be securely stored in compliance with institutional and regulatory requirements.

## Results

As of August 2025, data collection was complete, and we are analyzing data based on the protocol outlined earlier. Our CC-ACT workshops reached a total of 195 caregivers of individuals with NDDs, of whom 113 (57.9%) consented to participate in the research evaluation. Among these 113 participants, there were 4 (3.5%) dropouts or withdrawals from the study. In total, 3 focus groups were conducted, including 5 caregiver workshop participants, 13 facilitators, and 5 organizational leaders.

The team is in the middle of systematically analyzing these findings, with contributions from both researchers and individuals with lived experience. Qualitative survey and focus group data have undergone initial coding and are now being reviewed by the full team. Multiple data sources are being analyzed in parallel, each with dedicated leads: (1) RE-AIM framework implementation data; (2) participant outcome data, including self-reports and attendance records; (3) facilitator surveys and fidelity checklists; and (4) qualitative interview transcripts from caregivers, facilitators, and organizational leaders.

Our goal is to finalize all data analyses by the end of 2025, which will inform the preparation of best-practice guidelines (Table 4). Our analyses will help identify sociodemographic and process variables that moderate or mediate outcomes, helping to clarify the mechanisms contributing to improvements in psychological flexibility and resilience. Evaluation of the implementation processes using the RE-AIM framework will further illuminate the factors related to feasibility, acceptability, fidelity, adaptations, and resource use. These insights will help guide the broader adoption of the ACT intervention model across community settings.

**Table 4:** Project timeline.

Project activities	2022	2023	2024	2025
Engage, train, coach, prepare, and support implementation teams	✓			
Ethics approval	✓			
Implement first set of workshops	✓			
Implement second set of workshops	✓	✓		
Implementation evaluation	✓	✓	✓	
Conduct focus groups		✓		
Data analysis and knowledge translation		✓	✓	✓
Knowledge Translation and Exchange (forums, conferences, website, publications, and toolkit)		✓	✓	✓

## Discussion

### Anticipated Findings

Previous studies, including our own work, support the efficacy of CC-ACT to support the mental well-being of caregivers. This study will be important as there is a lack of effective evidence-informed interventions for caregivers of people with NDDs in Canada, and even fewer that have been studied in real-world settings. A meta-analysis found that ACT has significant effects on depressive and anxiety symptoms, stress, and quality of life in caregivers of various chronic conditions such as dementia, chronic pain, asthma, multiple sclerosis, and neurodevelopmental conditions [37]. Findings from this study will help to elucidate the practical implementation of this evidence-informed intervention across diverse settings in Canada, which may also pave the way for scale-up for caregivers of other chronic conditions as well.

All participating sites have successfully recruited and completed their planned workshops. As we formally analyze the data, we anticipate that we will gather nuanced implementation experiences under the RE-AIM framework.

In terms of reach, we will examine the sites’ challenges and successes in recruiting caregivers. Analysis of parameters, such as the preferred time of the year for the workshops, will inform how best to organize and plan caregiver interventions in the future. Furthermore, analysis of the HEIA data, conducted in phase 1, will be valuable in identifying regional and contextualized equity and access barriers, such as ethnicity, gender, SES, and language.

Our earlier work demonstrated the overall efficacy of our workshops among caregivers of people with autism, as implemented in the context of a hospital-based research study [14,16,17]. We anticipate similar positive results with the current protocol. However, we anticipate that the degree of effectiveness may vary with implementation in real-world contexts, for example, due to population differences as well as implementation fidelity. Given the diversity of workshop participants across sites, we expect to be able to identify some predictors of effectiveness (eg, education, ethnicity, and gender) and to examine whether different ACT processes mediate outcomes across participant subgroups. Follow-up data may also shed light on the factors associated with the individual maintenance of ACT skills.

Our evaluation of the implementation across the 11 sites will help us explore and understand the balance between fidelity and adaptation. We anticipate that the workshop fidelity will generally be high, as our intervention exercises have built in flexibility, and we have offered customized coaching to the facilitation teams. Consistent with Lake et al [38], we anticipate that the complementary perspectives of caregiver and clinician facilitators can enhance the experience of facilitator teams as well as the workshop.

With the structure and support provided by the study, all sites successfully adopted the workshop model. We anticipate that the analysis of feedback from site facilitation teams and organizational leadership will reveal factors that facilitate or hinder adoption, as well as identify what is needed for long-term maintenance beyond the study period. Throughout the study, we systematically documented the implementation processes and contextual factors at the participating sites. They will be analyzed to identify common facilitators, barriers, and effective delivery strategies. Insights will be synthesized into a set of evidence-informed best-practice guidelines to support future community-based workshop implementation. These guidelines will include recommendations on facilitator training, participant engagement, cultural and contextual adaptations, and resource planning, with the aim of enhancing scalability, fidelity, and sustainability across diverse community settings.

### Strengths and Limitations

One of the key strengths of our intervention workshop is its cofacilitation model, as workshops are delivered jointly by caregivers and clinicians. This approach grounds the intervention in lived experience, enhances participant engagement, and promotes relevance to caregivers. Our ACT exercises are uniquely experiential, allowing flexible in-session adaptation to participants’ needs while maintaining fidelity to core principles. Delivery across diverse geographical regions and organizations with varied cultures and challenges enables the examination of contextual factors that influence implementation.

During the study, we developed and refined a detailed protocol, fidelity checklist, and implementation supports (eg, handouts and email templates), as well as a flexible, peer-led coaching system. These supports facilitated the consistent delivery of the workshops in the study, as well as for future use, while providing an opportunity to evaluate the need and effectiveness of coaching in facilitating high-quality implementation. The use of a mixed methods design, guided by the RE-AIM framework, allows a comprehensive assessment of both intervention effectiveness and implementation processes.

This study also has several limitations. It was not conducted as an RCT, which limits causal inference, although RCTs are not always necessary in implementation research. We did not assess child outcomes, despite the potential indirect benefits for the children of participating caregivers—an important avenue for future research. Our outcomes relied on self-report measures, which may be subject to recall and social desirability bias. The study may also be limited by selection bias, as some caregivers might not have been reached by our study due to various systemic factors, and not all workshop participants consented to complete the research study. Although the HEIA tool was used to help identify potential gaps in accessibility, this study was not designed to address all identified issues. Limitations in site mandates, resources, and policies constrained our ability to adapt to certain groups; for example, targeting only English-speaking populations in Quebec due to the resource constraints for linguistic and cultural adaptations in this study.

### Next Steps and Future Directions

We have been collecting observations from coaches, facilitators, and workshop participants and have begun updating the program website accordingly. Once the final results are analyzed and published, the website will serve as a central platform for disseminating evidence-informed practices. Since 2015, our community of practice has grown to include over 250 individuals trained in the ACT Caring for the Caregiver programs. This network will be engaged in sharing information through the website, quarterly newsletters, and social media channels. Partner agencies, already actively involved in the project, will also contribute to dissemination efforts through presentations in their community. In addition, the findings will be presented at academic conferences and submitted for publication in peer-reviewed journals.

Our findings will not only inform future iterations of the ACT workshops but also guide best practices in scaling caregiver mental health programs, especially in virtual and hybrid formats. Understanding factors that facilitate or hinder implementation—such as populations not reached and systemic barriers to adopting the clinician-caregiver cofacilitation model—will be critical to ensuring the long-term sustainability and equitable reach of such interventions.

### Conclusions

This study aims to offer valuable insights into the real-world implementation of a caregiver-clinician cofacilitated ACT workshop across diverse communities in Canada. Embedding the intervention within the existing community agency infrastructures and tailoring delivery to local contexts will help ensure a practical and scalable approach to address caregiver mental health. As implementation continues across various sites, we anticipate that promising practices will emerge through local adaptations, coaching, and facilitator reflections.

Given the diversity in caregiver demographics and community needs, intervention supports and delivery methods must also be adapted to meet their unique needs. We expect that equity-related gaps will surface, particularly in relation to language, accessibility, and cultural relevance. These findings will be critical in informing future iterations of the intervention to ensure that it is not only effective but also inclusive and responsive to the diverse needs of caregiver communities. Through ongoing learning and refinement, we aim to contribute to more equitable, sustainable, and community-driven models of mental health support for families of individuals with NDDs.

## Data Availability

The datasets generated or analyzed during this study are not publicly available because the research participants did not consent to data sharing at the time of data collection, but are available from the corresponding author on reasonable request.

## Appendix

Multimedia Appendix 1 Semistructured interview guide for focus groups.

Multimedia Appendix 2 Peer review report by Kids Brain Health Network, Brain Canada Foundation, Health Canada.

## Abbreviations

ACT: acceptance and commitment training
CC-ACT: Caring for the Caregiver Acceptance and Commitment Training
FASD: fetal alcohol spectrum disorder
HEIA: Health Equity Impact Assessment
NDD: neurodevelopmental disability
RCT: randomized controlled trial
RE-AIM: reach, effectiveness, adoption, implementation, and maintenance
REDCap: Research Electronic Data Capture
