# Supplementary material for: Acceptance and Commitment Training for Family Caregivers of People with Neurodevelopmental Disabilities: Protocol for a Collaborative Implementation Study
Source: JMIR Res Protoc. 2025 Dec 4;14:e75049. doi: 10.2196/75049 (PMC12715472; doi:10.2196/75049)
Supplement: Multimedia Appendix 1 [file resprot_v14i1e75049_app1.docx]

**Appendix: Semi-Structured Interview Guide for Focus Groups**

**Introduction by Focus Group Facilitator**

**What is this focus group about:** Thank you for agreeing to participate in this focus group. This discussion will be about your experience in the workshop OR your experience co-facilitating/supporting the workshop. We would like to know what worked well or did not work well. Participation in the focus group is voluntary and you are free to stop/leave at any time. There are no right or wrong answers and you do not have to answer any questions that you do not want to answer. The focus group will last about 90-120 minutes.

**Audio recording details:** We will be recording the focus group so that we can transcribe what was said. The audio recording and transcriptions will be kept anonymous. You can leave the focus group at any time. Should you choose to withdraw after transcription, it will not be possible to identify and remove any comments you may made prior to withdrawing.

**Housekeeping items/rules for the focus group:** We ask that you respect the confidentiality of the group. Please do not record the focus group or share the link. Please keep all comments made during the focus group confidential and do not discuss what happened during the focus group outside the meeting. Your identity will be known only to the other focus group participants but we cannot guarantee that others in the group will respect the confidentiality of the group.

**Confirmation of participation:** If you would like to leave the focus group that would be okay to do before we get started. If you agree with the audio recording and the rules of the focus group, we can get started.

**Focus Group Questions for Facilitators and Leadership**
1) What made you interested in co-facilitating and/or hosting this workshop?

1. How did the ACT intervention model fit with the mandate of your organization/agency/hospital and with how you support family caregivers?
2. The workshop was co-facilitated by caregivers and clinicians.
   1. Is this partnered model new to your organization?
   2. What value do you see in the partnered model of caregivers and clinicians offering these workshops together?
3. Do you see the value/benefit in continuing to offer ACT workshops in the future? Why or why not?
4. How did the intervention reach the population in need/served by your organization?
5. How did the intervention reach the population in need not usually served by your organization?
6. The HEIA tool used in this project is intended to guide Equity, Diversity, and Inclusion (EDI) considerations in implementing the intervention, including the populations it reaches. Please comment on any EDI issues around the intervention and what can facilitate this.
7. What was your experience recruiting workshop participants?
   1. What helped you recruit participants?
   2. What challenges did you experience recruiting participants?
   3. What are the limitations in your recruitment strategy?
      1. Are there any other types of participants you would wish to reach?
      2. Is there anything you would do differently recruiting participants next time?
8. Is there anything else that you would like to talk about or share?

**Focus Group Questions for Facilitators Only (Breakout Room)**

1. What did you like about co-facilitating the workshop?
2. Based on your observations of the workshop participants, what is the value or benefits of the group to participants?
3. Was it feasible for you to co-facilitate the workshop?
   1. What helped?
   2. What did you find challenging (e.g., time, pay, personnel support)?
      1. How can these challenges be addressed?
4. How did the intervention fit with your personal values/beliefs?
5. How did the intervention fit with your clinical practice?
   1. Is ACT new to you/your practice?
   2. Do you use ACT with other clients?
6. Tell us about your experiences co-facilitating with a caregiver or clinician.
   1. What did you learn from or like about co-facilitating with a caregiver or clinician?
   2. What worked well?
   3. What was challenging?
7. What do you need to continue to co-facilitate these workshops over the long-term?
8. Did participants drop out of your workshop?
   1. If yes, why do you think people dropped out and how could you better support people to complete the workshop?
9. Did you make any changes to the workshop (content, structure, # of sessions). If yes, what changes did you make and why?

**Focus Group Questions for Leadership Only (Breakout Room)**

1. How feasible was it for your organization to offer the workshop?
   1. What helped?
   2. What got in the way?
2. What does your organization need to continue to offer these workshops over the long-term? (e.g., resources, funding, staff time, supports)
3. What, if any, challenges do you think exist in sustaining this caregiver-clinician partnership model within your organization?

**Focus Group Questions for Workshop Participants (Breakout Room)**

*Workshop Experience*

1. What did you like about the workshop?
2. What did you dislike about the workshop?
3. How did you feel about having caregivers and clinicians partner to lead the workshop?
4. How have you applied what you learned in the workshop to your life?
5. What would help sustain your ACT practice in the future?
6. What are your recommendations for future workshops?

*Workshop Logistics*

1. How did you hear about the workshop?
   1. Do you have recommendations on how to reach caregivers?
2. What made you interested in joining the workshop?
3. How feasible was it for you to attend and participate in the workshop?
   1. What helped?
   2. What got in the way?
   3. Was the format (virtual/in-person, time of day, # of sessions) acceptable/ preferable/ suitable to you?
4. What resources or supports do you think are needed to help caregivers attend future workshops?
5. These workshops were funded as part of a research project - do you see value in participating in research? Is there anything that made you hesitant to participate in research or about the research process?
6. Is there anything else that you would like to talk about or share?
