# Supplementary material for: Acceptance and Commitment Training for Family Caregivers of People with Neurodevelopmental Disabilities: Protocol for a Collaborative Implementation Study
Source: JMIR Res Protoc. 2025 Dec 4;14:e75049. doi: 10.2196/75049 (PMC12715472; doi:10.2196/75049)
Supplement: Multimedia Appendix 2 [file resprot_v14i1e75049_app2.pdf]

Research Program Reporting (Year 1)

Strategic Investment Fund
January 1, 2022-September 30, 2022 (Q1-Q3)

Program Title:
Implementation of a Collaborative ACT Intervention for Family Caregivers

Principal Investigator(s) (Institution)

Johanna Lake (Centre for Addiction and Mental Health)

Yona Lunsky (Centre for Addiction and Mental Health)

Kenneth Fung (University Health Network)

Submission Deadline October 15, 2022

PROJECT TITLE: IMPLEMENTATION OF A COLLABORATIVE ACT INTERVENTION FOR FAMILY CAREGIVERS

FUNDING TERM JANUARY 1, 2022 – DECEMBER 31, 2023

FUND DISTRIBUTIONS Y1: $90,310.94| Y2: $106,172.77 | Total Award: $196,483.71

Investigators/Partners and Project Roles:

	Name (include full credentials)	Primary Institution/Organization	NCE Funded
PI	Johanna Lake	Centre for Addiction and Mental Health	Yes
	Leading the project and coordinating with all the partners		
Co-PI	Yona Lunsky	Centre for Addiction and Mental Health	Yes
	Lead role in family engagement activities		
Co-PI	Kenneth Fung	University Health Network	Yes
	Evaluating the fidelity of the intervention, accountability for ensuring diversity and inclusion across project activities.		
Co-I	Jonathan Weiss	York University	Yes
	Expert in psychology and research design		
Co-I	Kendra Thomson	Brock University	
	Expert in psychology/behaviour analysis and coaching		
Co-I	Lee Steel	Centre for Addiction and Mental Health	
	Expert Family Leader and ACT coach		
Co-I	Melanie Penner	Holland Bloorview Kids Rehabilitation Hospital	
	Expert in developmental pediatrics and health services		
Co-I	Nicole Bobbette	Queen's University	
	Expert in occupational therapy and qualitative analysis		
Co-I	Jodie Siu	BC Centre for Ability	
	Expert Family Leader and ACT coach		
Partner	Scarborough Centre for Healthy Communities		
	Conduct 2 ACT workshops to caregivers of children, youth and adults with neurodevelopmental disabilities in the Scarborough region of Toronto		
Partner	Holland Bloorview Kids Rehabilitation Hospital		
	Conduct 2 ACT workshops to caregivers of children and youth with neurodevelopmental disabilities who are current or former Holland Bloorview clients.		
Partner	BC Centre for Ability		
	Conduct 2 ACT workshops to caregivers of children and youth with neurodevelopmental disabilities in BC		
Partner	Sibling Collaborative		
	Conduct 2 ACT workshops to sibling caregivers of transition age youth and adults with disabilities across Canada		
Partner	Bethesda Services		
	Conduct 2 ACT workshops to caregivers of children, youth and adults with neurodevelopmental disabilities in St. Catharine’s region		
Partner	Alberta Health Services		
	Conduct 1 (or 2) ACT workshop to caregivers of children with neurodevelopmental disabilities residing in Alberta		
Partner	Grandview Kids		
	Conduct 1 (or 2) ACT workshop to caregivers of children and youth with neurodevelopmental disabilities in the Durham region		

A. Project Lay Summary [For Web and Public Dissemination, ½ Page Max]:

Family caregivers of people with neurodevelopmental disabilities (NDDs) often experience stress and mental health difficulties (e.g., anxiety, depression); however, there are few services to support their wellbeing. Acceptance and

Commitment Training (ACT), which increases acceptance and mindfulness skills to help people cope, may be helpful to them. Our ACT-based intervention is unique in that it is co-designed and co-delivered by clinicians and caregivers together. We found that caregivers of people with autism and FASD who participated in our workshops reported feeling less depressed, stressed and isolated. We have started to train new caregivers and clinicians from across Canada to deliver ACT workshops, both in-person and virtually. An important next step is to support our newly trained facilitators to deliver ACT to caregivers of people with NDDs in their communities, and to better understand how it works when implemented in real-world conditions. We also want to understand what makes it easy or hard for caregivers and clinicians to co-lead the intervention together. These findings will help us successfully spread our partnered ACT intervention to other agencies and communities, and contribute to the movement of empowering caregivers to have their voices heard in co-creating, co-designing, and co-delivering interventions for other caregivers.

B. Y1 (Q1-Q3) Progress Report

B1. Project Activity and Achievement Summary (Q1-Q3): [3-pages max]

Objective 1

Evaluate the **implementation** of a caregiver and clinician-partnered ACT intervention delivered in communities across Canada to support the mental health and resilience of caregivers of individuals with NDD

A) **Evaluate whether the intervention leads to enhanced mental health and resilience in participants:** To date, we have delivered 2 ACT workshops (BC Centre for Ability and SCHC) to 14 caregivers of individuals with NDDs, and 3 workshops are either in the recruitment phase or are currently underway (HBKR, Siblings Canada and Bethesda Services). One of the original sites (Autism Nova Scotia) has encountered a number of barriers to participation and is withdrawing from this project. We have recruited two additional sites to replace them (Grandview Kids and Alberta Health Services). The new sites have started coaching and are on track to deliver their first workshops in January and February of 2023. A no cost extension, would allow these two teams time to complete two workshops as opposed to just one. The other 5 teams have scheduled their second set of workshops to roll out between January and April 2023. Across the two teams who have completed their first workshop, 12 of the 14 workshop participants consented to the research, and 6 completed all three data collection time points. Recruiting participants to engage in the research evaluation has been challenging - we recruited fewer participants than anticipated (4 at one site and 8 at the other) and we also had fewer participants complete all data collection time points (9 completed the pre-survey, 8 the post-survey, and 7 the follow-up survey across the two sites). Some of these challenges were related to REB delays which meant we had less time for research recruitment, as well as demands and pandemic-related stressors experienced by both facilitators and workshop participants. For one team their workshop participant numbers were quite small which also meant we had fewer participants to recruit from (N=10). To mitigate these challenges we: a) created a video explaining why research is important from the perspective of a caregiver and researcher; b) encouraged teams to host a workshop orientation session prior to the start of their workshop where we can join to share information about the research evaluation and answer participant questions; c) highlighted the importance of mentioning the research during workshop recruitment screening calls; and d) allowed participants to join the research up to the start of the second workshop session.

B) **Assess feasibility of recruiting caregivers for the intervention and percentage of participants who complete the workshop**: Of the two teams who completed their workshops thus far, both were able to recruit enough caregivers to run the groups. However, both teams noted that recruitment took longer and required more time and resources than they anticipated. For one team, they also experienced considerable attrition prior to the workshop starting and once it was underway (4 participants dropped out). To mitigate these challenges, facilitators plan to: a) recruit administrative support to help; b) ask leadership for more time to support workshop recruitment; c) host an orientation session to explain the intervention to a large group of people; d)start recruitment sooner; and e) recruit more workshop participants given attrition

C) **Evaluate perceptions and experiences of intervention by participants, clinician facilitators and caregiver facilitators**: The 5 facilitators who facilitated a workshop (2 teams) thus far, have each completed the facilitator experiences survey which asks questions about their experience and perception of the intervention. In addition, facilitators have been sharing their experiences so far during our quarterly team meetings and coaching sessions. Workshop participants who consented to the research have also been sharing their perceptions and experiences receiving the intervention through our pre, post and follow-up surveys. We anticipate learning more about facilitators' experiences during the focus groups planned in Year 2, as well as when each team launches their second workshop and subsequent evaluations. Please see above strategies to help mitigate challenges associated with workshop participant research recruitment.

D) **Assess what adaptations become necessary as the intervention is implemented in various real-world settings:** Each of the two teams who completed their workshops also completed their session fidelity checklists. We anticipate learning more about what adaptations become necessary as subsequent teams complete their fidelity checklists, as well as during the focus groups and internal stakeholder forums planned in Year 2.

Objective 2

Evaluate the **unique collaboration between caregivers and clinicians** co-delivering the intervention

A) **Identify the barriers and facilitators to successful caregiver-clinician collaborations with regard to facilitator training and intervention delivery:** All 5 facilitators who facilitated a workshop (2 teams) completed the facilitator experiences survey which asks about the barriers and facilitators to successful caregiver-clinician collaborations in the context of delivering the workshop. We anticipate learning more about the barriers and facilitators from various stakeholders perspectives during the focus groups planned in Year 2, as well as when each team launches their second workshop and subsequent evaluations. One team struggled to come together as a caregiver-clinician team; particularly in terms of the clinicians on the team finding the time to meet as a group and recognizing the importance of doing this. This team also lost one clinician facilitator, who was no longer able to facilitate before the workshop began. To mitigate this we: a) debriefed with the teams (we will also offer this type of facilitated debriefing session to other teams as needed) after their workshop to explore what went well/what was challenging about the collaboration and what could be improved for their next workshop from each facilitator's perspective; b) encouraged teams to make time not only to practice and prep, but also to connect as a team prior to workshops; c) supported teams in preparing for their workshops well in advance so there is time to schedule meetings and fewer time pressures/constraints; d) established a virtual ACT community of practice based on feedback from our partners to share, problem-solve and build ACT skills; and e) as needed, some facilitators have advocated through their organizations for more time to prepare for the workshops.

B) **Assess the value of this collaboration from various stakeholders' perspectives, including participants, clinician facilitators and caregiver facilitators**: All 5 facilitators who facilitated a workshop (2 teams) completed the facilitator experiences survey which asks questions about the value of this collaboration from the perspective of facilitators. Workshop participants who consented to the research have also been sharing their perceptions of this collaboration through our post survey. We anticipate learning more about the value of this collaboration from various stakeholders perspectives during the focus groups planned in Year 2, as well as when each team launches their second workshop and subsequent evaluations. Please see above strategies to help mitigate challenges associated with workshop participant research recruitment.

B2. Response to Administrative Recommendations [2-pages max]

Recommendations:

1. HQP engagement not directly addressed
2. Minor issue the end-of-funding KT was mentioned in the proposal, but the plan was not well described (e.g., who and how will this facilitate wider implementation of program).
3. EDI statement was mostly appropriate, but a bit generic, did not provide compelling ways to integrate/incorporate marginalized groups/communities.

4. No mention of pandemic-related stresses in the home, which may be exceptional and distinct from pre-pandemic caregiver/family issues.

Response:

1. Depending on their role in the project, HQP will assist with data analysis and manuscript writing, toolkit development, and sharing findings with relevant audiences. HQP will also assist with survey administration, conducting focus groups, website development/maintenance, and planning of both the internal and external forums.
2. We have further described our end of grant KT plan. In the summer of 2023, we will host an internal stakeholder forum where we will bring together different stakeholder groups to share and discuss: a) preliminary project findings around the impact of the workshops, b) the experiences of facilitators in the co-delivery model, and c) barriers and facilitators to the implementation of ACT In the community. This forum will also involve updates from each of our sites around lessons learned, which will be used to inform our ACT implementation toolkit. The external forum will include stakeholders from outside of the project/the community and will be an opportunity to share our project findings, including a finalized version of the toolkit. In the summer of 2022 four members of our team attended and presented project findings at an international conference (DOHaD). Our team has also been sharing project findings thus far with relevant community, academic and clinical audiences.
3. We have adapted and implemented the Health Equity Impact Assessment (HEIA) tool with each of our sites to help ensure equitable and inclusive recruitment and delivery of our workshops. EDI-related issues are discussed with each team during coaching sessions and at our quarterly team meetings, as well as internal and external stakeholder forums. EDI as it pertains to implementing ACT in the community will be described in our ACT implementation toolkit.
4. We have adapted some of our pre, post and follow-up questions to address pandemic-specific stresses that caregivers may be experiencing. We will also be tracking the time when the workshops are offered so that we can correlate with COVID-related waves or restrictions in particular regions.

B3. Partnerships: [1-page max]

Table 1. Description of partner agencies

Partner Agency	Role	# of Trained Facilitators	Changes in Partnerships during Project Term
Scarborough Centre for Healthy Communities (Ullanda Niel)	-Receive coaching facilitator support leading up to and once ACT workshops underway<br>-Complete HEIA tool (before workshop), fidelity checklist (once workshop underway), and facilitator reflections survey (after workshop has ended)<br>-Participate in focus groups in Year 2<br>-Provide two ACT workshops to caregivers of children, youth and adults with neurodevelopmental disabilities in a regional (urban, inner-city) context.	2 clinicians & 2 caregivers	a new caregiver will co-facilitate the second workshop
BC Centre for Ability (Sacha Bailey)	-Receive coaching facilitator support leading up to and once ACT workshops underway	1 caregiver & 1 clinician	n/a

	-Complete HEIA tool (before workshop), fidelity checklist (once workshop underway), and facilitator reflections survey (after workshop has ended)<br>-Participate in focus groups in Year 2<br>-Provide two ACT workshops to caregivers of children and youth with neurodevelopmental disabilities in BC		
Siblings Canada (Helen Ries)	-Receive coaching facilitator support leading up to and once ACT workshops underway<br>-Complete HEIA tool (before workshop), fidelity checklist (once workshop underway), and facilitator reflections survey (after workshop has ended)<br>-Participate in focus groups in Year 2<br>-Provide two ACT workshops to sibling caregivers of transition age youth and adults with disabilities across Canada	1 clinician/caregiver & 2 caregivers	n/a
Bethesda Services (Linda Moroz)	-Receive coaching facilitator support leading up to and once ACT workshops underway<br>-Complete HEIA tool (before workshop), fidelity checklist (once workshop underway), and facilitator reflections survey (after workshop has ended)<br>-Participate in focus groups in Year 2<br>-Provide two ACT workshops to caregivers of children, youth and adults with neurodevelopmental disabilities in the Niagara region (rural)	2 clinicians & 1 caregiver	n/a
Holland Bloorview Kids Rehabilitation Hospital (Sheelagh Jamieson)	-Receive coaching facilitator support leading up to and once ACT workshops underway<br>-Complete HEIA tool (before workshop), fidelity checklist (once workshop underway), and facilitator reflections survey (after workshop has ended)<br>-Participate in focus groups in Year 2<br>-Provide two ACT workshops to caregivers of children and youth with neurodevelopmental disabilities who are current or past HBKR clients (tertiary, urban)	3 clinicians & 2 caregivers	n/a
Dalhousie Department of Family Medicine with Autism Nova Scotia (Catherine Rahey)		4 clinicians & 4 caregivers	**This partner is no longer part of this project - they needed to start later and cannot commit beyond a single workshop at this time. They are**

			**continuing with this work at a slower pace, with alternate funding (and an adult focus).**
Grandview Kids (Stephanie Stoddard)	-Receive coaching facilitator support leading up to and once ACT workshops underway<br>-Complete HEIA tool (before workshop), fidelity checklist (once workshop underway), and facilitator reflections survey (after workshop has ended)<br>-Participate in focus groups in Year 2<br>-Provide one or two ACT workshops to caregivers of children and youth with neurodevelopmental disabilities residing in the Durham region	1 clinician, 1 clinician/caregiver & 1 caregiver	**This partner was added to support the implementation of one or two ACT workshops in the Durham region (two workshops depending on whether NCE extension is approved).**
Alberta Health Services (Kesa Severtson)	-Receive coaching facilitator support leading up to and once ACT workshops underway<br>-Complete HEIA tool (before workshop), fidelity checklist (once workshop underway), and facilitator reflections survey (after workshop has ended)<br>-Participate in focus groups in Year 2<br>-Provide one or two ACT workshops to caregivers of children and youth with neurodevelopmental disabilities residing in the Durham region	1 clinician and 1 caregiver	**This partner was added to support the implementation of one or two ACT workshops in the Alberta region (two workshops depending on whether NCE extension is approved).**

B4. Project Deliverables/Outputs: [2-pages max]

List and briefly describe any:

Category	Project Activity or Product	Title	Attendance (If applicable)	Key Objectives & Outcomes
Stakeholder/ Community engagements	Developmental Origins of Health and Disease (DOHaD) Conference August 30, 2022	Presented poster titled “Family Engagement in Acceptance and Commitment Training: A Caregiver-Clinician Partnership Model”	100 (researchers, clinicians, community members, trainees) - estimated based on the number of people who came by the poster	Disseminate findings about the impact of our family engagement ACT model on caregiver and clinician facilitators in terms of: a) training to deliver the intervention; b) receiving coaching support; and c) co-facilitating the intervention. We also explored the impact of the caregiver-clinician partnered model on participants receiving the intervention.

Stakeholder/ Community engagements	KBHN Research Day Event (Aug 28, 2022)	Family Engagement in Acceptance and Commitment Training: A Caregiver-Clinician Partnership Model	50 people (researchers, clinicians, family members, leadership, network members)	Introduce and share project updates and obtain feedback from network members
Stakeholder/ Community engagements	CanChild's Luke's Legacy Family Research Rounds	Enhancing Caregiver Wellbeing: A Collaborative Acceptance and Commitment Training (ACT) Intervention	25 people (family members, researchers, clinicians)	Share about our collaborative ACT intervention model, including what we are learning from workshop participants and facilitators, as well as where we are headed next
Stakeholder/ Community engagements	CAMH Psychology Residents	Enhancing Caregiver Wellbeing: A Collaborative Acceptance and Commitment Training (ACT) Intervention	10 people (CAMH psychology residents)	Share about our collaborative ACT intervention model, including what we are learning from workshop participants and facilitators, as well as next steps/future directions.
Stakeholder/ Community engagements	CAMH Grand Rounds	Enhancing Caregiver Wellbeing: A Collaborative Acceptance and Commitment Training (ACT) Intervention	130 people (clinicians, researchers, and trainees at CAMH)	Share about our collaborative ACT intervention model, including what we are learning from workshop participants and facilitators, as well as next steps/future directions
Stakeholder/ Community engagements	Holland Bloorview Autism Research Centre Team Meeting	Enhancing Caregiver Wellbeing: A Collaborative Acceptance and Commitment Training (ACT) Intervention	10 people (clinicians, researchers and trainees at Holland Bloorview)	Share about our collaborative ACT intervention model, including what we are learning from workshop participants and facilitators, as well as next steps/future directions
Stakeholder/ Community engagements	Project Launch Meeting (February 2, 2022)		20 project team members and partner organization members (e.g., facilitators and stakeholders)	Provide an overview of the project, introduce team members and outline project roles and timelines
Stakeholder/ Community engagements	1st Project Quarterly Team Meeting (May 9,2022)		27 project team members and partner organization members (e.g., facilitators and stakeholders)	Share and exchange project/team updates, discuss lessons learned so far, and review next steps/timelines

Stakeholder/ Community engagements	2nd Project Quarterly Meeting (Sept 12, 2022)		25 project team members and partner organization members (e.g., facilitators and stakeholders)	Share and exchange project/team updates, discuss lessons learned so far, and review next steps/timelines
Physical/virtual products developed	Adapted HEIA tool for usability and to better reflect ACT workshop context	Adapted HEIA Tool	n/a	Promote equitable delivery of ACT intervention
Training Materials/Meetings	HEIA Meetings (March 30, 2022 & July 27, 2022)		18 trained caregiver and clinician co-facilitators (across the two meetings)	Introduce/explain the tool and its relevance to this project, as well as support completion of the tool
Stakeholder/ Community engagements	ACT Community of Practice (June 8, and September 7, 2022)		30 (15 per meeting) people (trained caregiver and clinician facilitators)	Problem-solve, share and discuss ACT-related questions, concerns, and tricky situations, as well as ACT best practices, resources and new knowledge

C. Project Management Table

Please use high level bullet format where possible, to limit text in this table and highlight (in **RED** font) those Y1 objectives/activities/deliverables that have shifted toward completion in subsequent terms (Y2).

A - Objectives	B – Annual Objectives		C – Annual Activities		D –Outputs/Deliverables		E -Challenges	F - Outcomes
**Program Objectives**	**Year 1**<br>**Planned/Achieved Objectives**	**Year 2**<br>**Planned Objectives**	**Year 1**<br>**Achieved Activities**	**Year 2**<br>**Planned Activities**	**Year 1**<br>**Achieved Deliverables**	**Year 2**<br>**Anticipated Deliverables**	**Challenges to Achieving Annual Deliverables Mitigations/Adaptations**	**Final Project Deliverables & Outcomes**
**Study 1 Objective 1**<br>Evaluate the **implementation** of a caregiver and clinician-partnered ACT intervention delivered in communities across Canada to support the mental health and resilience of caregivers of individuals with NDDs.	-Evaluate whether the intervention leads to enhanced mental health and resilience in participants<br><br>-Assess feasibility of recruiting caregivers for the intervention and percentage of participants who complete the workshop<br><br>-Evaluate perceptions and experiences of intervention by participants, clinician facilitators and caregiver facilitators	-Evaluate whether the intervention leads to enhanced mental health and resilience in participants<br><br>-Assess feasibility of recruiting caregivers for the intervention and percentage of participants who complete the workshop<br><br>-Evaluate perceptions and experiences of intervention by various stakeholders, including participants, clinician	-Delivered 2 ACT workshops (BC Centre for Ability and SCHC) to 14 caregivers of individuals with NDDs<br>-3 ACT workshops underway (HBKR, Siblings Canada and Bethesda)<br><br>-Provided coaching support to 7 teams<br><br>-12 workshop participants consented to the research	-Conduct 3 cross-site focus groups and 2 mixed focus groups - transcribe & analyze<br><br>-Deliver 7 ACT workshops to caregivers of individuals with NDDs<br><br>-Provide/offer coaching support to 7 teams as needed<br><br>-Analyze impact of workshop through pre, post and	-Shared preliminary project findings at an international conference (DOHaD) and through 6 presentations to a variety of academic and community audiences (i.e.., CAMH Grand Rounds, Luke's Legacy)<br><br>-Coached 6 of 7 partner teams (N=6) before launching their first workshop<br><br>-5 of 7 ACT workshops delivered or underway to caregivers of people with NDDs	-Finalize ACT Implementation Toolkit<br><br>-Disseminate findings and our toolkit with other interested agencies and policy makers (KTE event)<br><br>-Publish project findings through open access publications<br><br>-Share findings at a relevant international conference as well as with community stakeholders	-Recruiting participants to engage in the research evaluation has been challenging. To mitigate this we have: a) created a video explaining why the research is important from the perspective of a caregiver and researcher; b) joined workshop orientation sessions to share about the research and answer participant questions; c) highlighted the importance of mentioning the research during workshop recruitment screening calls; d)	-Improve the resilience, mental health and wellbeing of family caregivers of people with NDDs (n=168) from across Canada.<br><br>-Provide evidence of the impact of our ACT intervention and help identify sociodemographic and process variables that moderate or mediate that impact<br><br>-Identify factors relevant to the fidelity, feasibility, and

A - Objectives	B – Annual Objectives		C – Annual Activities		D –Outputs/Deliverables		E -Challenges	F - Outcomes
Program Objectives	Year 1<br>Planned/Achieved Objectives	Year 2<br>Planned Objectives	Year 1<br>Achieved Activities	Year 2<br>Planned Activities	Year 1<br>Achieved Deliverables	Year 2<br>Anticipated Deliverables	Challenges to Achieving Annual Deliverables Mitigations/Adaptations	Final Project Deliverables & Outcomes
	-Assess what adaptations become necessary as the intervention is implemented in various real-world settings<br><br>-	facilitators, caregiver facilitators, and organizational leaders<br><br>-Assess what adaptations become necessary as the intervention is implemented in various real-world settings<br><br>-Identify what demographic, clinical, and organizational variables predict or moderate change in workshop participants<br><br>- Assess long-term sustainability of the intervention program	(across two sites) and 6 completed all three data collection time points<br><br>-2 teams completed fidelity checklists<br><br>-4 teams completed HEIA survey<br><br>-5 facilitators completed facilitator experiences survey<br><br>-Held 1 project launch meeting and 2 quarterly team meetings	follow-up data obtained from workshop participants<br><br>-Analyze facilitator experiences survey data<br><br>-Analyze and interpret fidelity checklist data<br><br>-Analyze and interpret HEIA survey data<br><br>-Co-develop ACT Implementation Toolkit with partners<br><br>-Hold 1 quarterly team meeting			allowed participants to join the research up to the second workshop session<br><br>-Facilitators have experienced time and resource challenges recruiting workshop participants. To mitigate this facilitators have: a) recruited administrative support to help; b) asked for additional work time to support workshop recruitment; c) hosted an orientation session as a more efficient way to explain the workshop to a large group of people<br><br>-The HEIA tool in its original form	acceptability of the intervention in the community.<br><br>-Build capacity and skills of ACT caregiver and clinician facilitators from across Canada<br><br>-Disseminate our ACT caregiver-clinician intervention model through the co-development of an ACT implementation toolkit and external KTE forum

A - Objectives	B – Annual Objectives		C – Annual Activities		D –Outputs/Deliverables		E -Challenges	F - Outcomes
**Program Objectives**	**Year 1**<br>**Planned/Achieved Objectives**	**Year 2**<br>**Planned Objectives**	**Year 1**<br>**Achieved Activities**	**Year 2**<br>**Planned Activities**	**Year 1**<br>**Achieved Deliverables**	**Year 2**<br>**Anticipated Deliverables**	**Challenges to Achieving Annual Deliverables Mitigations/Adaptations**	**Final Project Deliverables & Outcomes**
			-Shared preliminary projects findings at DOHaD conference	-Host internal stakeholder forum bringing together our team of facilitators, family leaders, participants, and organizational leaders to review preliminary data and begin to develop KT tools and resources<br><br>-Host KTE event external stakeholder forum			was not easily completed by facilitators and the time to complete it was onerous. To mitigate this we: a) hosted 2 HEIA meetings (March 30, 2022; N= 8 and July 27, 2022 N=10) to explain the tool and its relevance to this project; b) adapted the tool survey based on facilitator feedback to simplify the language and contextualize to our ACT workshops; c) offered to complete the tool with our support instead of having teams complete it independently; d) asked teams complete the tool once rather than twice; e) provided a summary of each team's survey responses to share	

A - Objectives	B – Annual Objectives		C – Annual Activities		D –Outputs/Deliverables		E -Challenges	F - Outcomes
**Program Objectives**	**Year 1**<br>**Planned/Achieved Objectives**	**Year 2**<br>**Planned Objectives**	**Year 1**<br>**Achieved Activities**	**Year 2**<br>**Planned Activities**	**Year 1**<br>**Achieved Deliverables**	**Year 2**<br>**Anticipated Deliverables**	**Challenges to Achieving Annual Deliverables Mitigations/Adaptations**	**Final Project Deliverables & Outcomes**
							back and reflect on as a team; f) asked the teams to focus on one population rather than multiple populations to reduce completion time<br><br>-Some facilitators have reflected that the time spent in coaching has been burdensome. To mitigate this we: a) recorded coaching meetings for facilitators who were unable to attend live sessions; b) reminded teams that coaching can be tailored to each team's needs and availability; c) started coaching well in advance of workshops so as to space out sessions over a longer period of time; d) reminded teams to	

A - Objectives	B – Annual Objectives		C – Annual Activities		D –Outputs/Deliverables		E -Challenges	F - Outcomes
**Program Objectives**	**Year 1**<br>**Planned/Achieved Objectives**	**Year 2**<br>**Planned Objectives**	**Year 1**<br>**Achieved Activities**	**Year 2**<br>**Planned Activities**	**Year 1**<br>**Achieved Deliverables**	**Year 2**<br>**Anticipated Deliverables**	**Challenges to Achieving Annual Deliverables Mitigations/Adaptations**	**Final Project Deliverables & Outcomes**
							use the stipend to offset time spent outside of work hours; e) advocated with leadership to allocate more time for coaching during regular working hours	
**Study 1 Objective 2**<br>Evaluate the **unique collaboration between caregivers and clinicians** co-delivering the intervention	-Identify the barriers and facilitators to successful caregiver-clinician collaborations with regard to facilitator training and intervention delivery<br><br>-Assess the value of this collaboration from various stakeholders' perspectives,	-Identify the barriers and facilitators to successful caregiver-clinician collaborations with regard to facilitator training and intervention delivery<br><br>-Assess the value of this collaboration from various stakeholders' perspectives,	-5 facilitators completed facilitator experiences survey	-Conduct 3 cross-site focus groups and 2 mixed focus groups - transcribe & analyze<br><br>-Analyze facilitator experiences survey data<br><br>-Co-develop ACT Implementatio	-Shared preliminary project findings at an international conference (DOHaD) and through 6 presentations to a variety of academic and community audiences (i.e.., CAMH Grand Rounds, Luke's Legacy)	-Finalize ACT Implementation Toolkit<br><br>-Disseminate findings and our toolkit with other interested agencies and policy makers (KTE event)<br><br>-Publish project findings through open access publications	-One team has struggled to come together as a team; both in terms of recognizing the importance of investing in this time and having the time to do it. This team also lost one clinician facilitator, who was no longer able to facilitate before the workshop began. To mitigate this we: a) debriefed with the team after their	-Ascertain information about the perceived value, barriers and facilitators to caregiver-clinician collaborations, from multiple stakeholder perspectives.<br><br>-Promote caregiver-clinician

A - Objectives	B – Annual Objectives		C – Annual Activities		D –Outputs/Deliverables		E -Challenges	F - Outcomes
**Program Objectives**	**Year 1**<br>**Planned/Achieved Objectives**	**Year 2**<br>**Planned Objectives**	**Year 1**<br>**Achieved Activities**	**Year 2**<br>**Planned Activities**	**Year 1**<br>**Achieved Deliverables**	**Year 2**<br>**Anticipated Deliverables**	**Challenges to Achieving Annual Deliverables Mitigations/Adaptations**	**Final Project Deliverables & Outcomes**
	including participants, clinician facilitators and caregiver facilitators,	including participants, clinician facilitators, caregiver facilitators, organizational leaders, and the research team		n Toolkit with partners		-Share findings at a relevant international conference as well as with community stakeholders	workshop to explore what went well/what was challenging in co-facilitating and what could be improved for their next workshop; b) encouraged teams to make time not only to practice and prep, but also to connect as a team; c) facilitators from this team advocated through their organizations for more time and support (administrative support to help with recruitment) to prepare for the workshops	collaborations beyond ACT<br><br>-Disseminate our ACT caregiver-clinician intervention model through the co-development of an ACT implementation toolkit and external KTE forum

SIF09 ACTProgram **Johanna Lake**

Community Implementation and Evaluation of Acceptance and Commitment Training (ACT) for Family Caregivers

Review Panel Results: **Invitation to Apply**

REVIEWER 1

Project Overview:

The proposed project aims to evaluate the feasibility, acceptability and perceived effectiveness of implementing a community-partnered Acceptance and Commitment Training (ACT) intervention to support the mental health and wellbeing of families of individuals with NDDs. The project will also evaluate the barriers and facilitators of caregivers and clinicians implementing this intervention together. The context of the Covid-19 pandemic is considered and the project will explore both in-person and online methods of the specific intervention. This will prepare the ground for a more rigorous RCT to test actual effectiveness.

Evaluation/score comments (provide brief comments for each criterion identifying strengths/weaknesses):

Strengths: This is an important project addressing the needs of caregivers and families of individuals with neurodevelopmental disorders. The evidence supporting the proposed ACT intervention are solid. The context of the pandemic is taken into consideration, leading to online adaptations of previously proven interventions. Very strong research team and family engagement plan. Study seems feasible. Strong potential for sustainability beyond grant. Very strong support from multiple partners (funders and service providers from across country).

Weaknesses: Main weakness is that the proposed project seems to be already embedded in an ongoing program of research that is well funded from multiple other sources. Depending on how KBHN perceives this, this might be an opportunity to build on a strong foundation of funded work; or it could be an example of leveraging without much 'new' work happening.

REVIEWER 2

Project Overview:

The objective is to expand implementation of the ACT intervention, virtually and in-person, across Canada, and evaluate caregiver participant outcomes, and the experience of teams of caregivers and clinician facilitators. The Acceptance and Commitment Training aims at enhancing mental health support for families of individuals with NDDs.

They will perform a randomized effectiveness trial. They will assess caregiver distress, social isolation, self-compassion, depression, perceived health and the parent-child relationship, before, immediately after, and 3 months post-workshops. They will also evaluate the barriers.

Strengths:

Organization and support. Advancements already performed.

Weaknesses:

Some variables missing in the evaluation plan, including time since diagnosis, proximity to important life events etc…

REVIEWER 3

Strengths:

-builds from established intervention program

-implementation focus

-strong family engagement in the project

-strong partnerships involving service delivery organizations in 3 provinces (sustainability)

-established funding from Azrieli Foundation

-consistent with many of KBHN's programs that directly engage parents/caregivers in the intervention

Weaknesses: None Identified

Partner Letters of Support

Strengths:

Prior partnerships with Bethesda, Holland Bloorview, Azrieli
We will work with our community partners to recruit interested caregiver participants and coach family caregiver and clinician facilitators

Azrieli

- LOS - $250,000 of Azrieli funding will be dedicated to work on this project [LOI says $220K]
- Host project resources on website; disseminate project outputs, share knowledge through Communications team, assist with recruitment of participants for workshops, advise on engagement

Holland Bloorview:

- Involve the 14 clinicians and 6 family members who previously participated in ACT training

- Commit to running and evaluating the impact of at least two ACT workshops for caregivers of our clients, …across our hospital…
- Disseminate project outputs

Bethesda

- Involve two Bethesda clinicians
- Commit to running and evaluating the impact of at least two ACT workshops …. across our organization…
- Disseminate project outputs

Moving forward: they have worked together in the past and this seems to be an expansion

Weaknesses: none identified

Family Engagement

Strengths:

The ACT workshops have been co designed and delivered by family advisors. Two family advisors are listed who have worked with this research team for the past 8 years. Through prior research the team family caregivers offered ACT workshops with family caregivers and providers co leading these workshops. This co design and delivery method will continue within this proposal in the implementation of the study. Family members are listed as leaders and trainees within the project team. One family member and a graduate trainee (unclear if they are a family member of researcher) also participated in the family engagement in research course to build capacity in building family partnerships in research.

Weaknesses:

It is mentioned that family advisors co designed and delivered the ACT workshops. It is unclear if there are more than 2 advisors and their role in the overall project beyond designing and delivery of the workshops. Family members do not appear to be partners in the analysis or evaluation of the project. With an expansion to NDD and agencies across Canada, is there an opportunity to build more capacity with more family advisor involvement.
